# Draft Genome Sequence of *Lactobacillus paracasei* DUP 13076, Which Exhibits Potent Antipathogenic Effects against *Salmonella enterica* Serovars Enteritidis, Typhimurium, and Heidelberg

**DOI:** 10.1128/genomeA.00065-18

**Published:** 2018-02-15

**Authors:** Muhammed Shafeekh Muyyarikkandy, Fahad H. Alqahtani, Ion Mandoiu, Mary Anne Amalaradjou

**Affiliations:** a Department of Animal Science, University of Connecticut, Storrs, Connecticut, USA; b Computer Science and Engineering Department, University of Connecticut, Storrs, Connecticut, USA

## Abstract

*Lactobacillus paracasei* DUP 13076 demonstrates antagonistic effects against the foodborne pathogens *Salmonella enterica* serovars Enteritidis, Typhimurium, and Heidelberg in coculture and *in vitro* experiments. Here, we report the draft genome sequence of *Lactobacillus paracasei* DUP 13076, which has a circular chromosome of 3,048,314 bp and a G+C content of 46.3%.

## GENOME ANNOUNCEMENT

*Lactobacillus paracasei* is a Gram-positive rod-shaped nonmotile bacterium. It is a normal inhabitant of the human and animal gut microbiome and is extensively used in food production, particularly in the dairy industry as a starter culture (1). Additionally, several of the *L. paracasei* strains have been widely researched for their probiotic potential (1–3). Besides their ability to promote human health through anti-inflammatory and immunomodulatory properties, *L. paracasei* strains are also known to be effective in controlling pathogens, including *Candida albicans*, *Escherichia coli*, *Shigella dysenteriae*, *Cronobacter sakazakii*, and *Staphylococcus aureus* (4, 5). Similar to these strains, *L. paracasei* DUP 13076 was recently demonstrated to possess antimicrobial properties against *Salmonella enterica*. Our results revealed that *L. paracasei* DUP 13076 inhibited *Salmonella* adhesion and invasion in primary chicken cecal epithelial cells and survival in chicken macrophages by attenuating the key virulence genes required for pathogen colonization (6).

Genome sequencing of *L. paracasei* DUP 13076 was performed to identify specific genetic features of this strain and to elucidate its probiotic potential. Briefly, *L. paracasei* DUP 13076 was grown in de Man-Rogosa-Sharpe broth at 37°C for 24 h prior to genomic DNA extraction. The DNA was quality checked, and a paired-end library was generated using the Illumina MiSeq platform in the Microbial Analysis, Resources, and Services Facility at the University of Connecticut (Storrs, CT, USA). The average insert size and read lengths were 550 bp and 251 bp, respectively. The quality check was performed using FastQC version 0.11.5 (http://www.bioinformatics.babraham.ac.uk/projects/fastqc/). Adaptors, primers, and terminal bases with a Phred score of <20 were trimmed using Trimmomatic version 3.10.1 (7). The SPAdes genome assembler version 3.10.1 (8) was used for *de novo* assembly, and the outputs were quality checked with QUAST version 3.1 (9). Genome annotations were carried out using the Rapid Annotations using Subsystems Technology (RAST) server (10) and the NCBI Prokaryotic Genome Automatic Annotation Pipeline (PGAAP) (11), and the results were combined.

The assembled draft genome of* L. paracasei* DUP 13076 revealed that it has a circular chromosome of 3,048,314 bp with a G+C content of 46.3%. There were 150 contigs identified, with an average length of 153,320 bp, and the largest contig size was 434,880 bp. The chromosome contains 342 subsystems with a coding sequence for 3,066 genes. The NCBI Prokaryotic Genome Annotation Pipeline predicted 77 RNA genes (16 rRNAs, 58 tRNAs, and 3 noncoding RNAs [ncRNAs]). Subsystem feature counts indicated that a majority of the genes classified within subsystems were associated with cellular metabolism (76%), followed by cell wall and capsule synthesis (7%) and membrane transport (4%). Additionally, genes involved in polyamine, betaine, and glycine synthesis and uptake were recognized. Further, genes with potential probiotic attributes, including those for adhesion and the colicin V and bacteriocin cluster, were also identified. No remarkable virulence-associated genes were found.

### Accession number(s).

This whole-genome shotgun project has been deposited at DDBJ/ENA/GenBank under the accession number PKQJ00000000. The version described in this paper is version PKQJ01000000.
